# Molecular and Cellular Effects of *In Vitro* Shockwave Treatment on Lymphatic Endothelial Cells

**DOI:** 10.1371/journal.pone.0114806

**Published:** 2014-12-11

**Authors:** Sabrina Rohringer, Wolfgang Holnthoner, Matthias Hackl, Anna M. Weihs, Dominik Rünzler, Susanna Skalicky, Michael Karbiener, Marcel Scheideler, Johannes Pröll, Christian Gabriel, Bernhard Schweighofer, Marion Gröger, Andreas Spittler, Johannes Grillari, Heinz Redl

**Affiliations:** 1 Ludwig Boltzmann Institute for Experimental and Clinical Traumatology, Donaueschingenstrasse 13, Vienna, Austria; 2 Austrian Cluster for Tissue Regeneration, Vienna, Austria; 3 Department of Biochemical Engineering, University of Applied Sciences Technikum Wien, Hoechstaedtplatz 6, Vienna, Austria; 4 TAmiRNA GmbH, Muthgasse 11, Vienna, Austria; 5 Institute for Molecular Biotechnology, Graz University of Technology, Petersgasse 14, Graz, Austria; 6 Red Cross Blood Transfusion Service, Krankenhausstrasse 7, Linz, Austria; 7 Skin and Endothelium Research Division, Department of Dermatology, Medical University of Vienna, Vienna, Austria; 8 Core Facility Imaging, Medical University of Vienna, Vienna, Austria; 9 Core Facility Flow Cytometry & Surgical Research Laboratories, Medical University of Vienna, Vienna, Austria; 10 Department of Biotechnology, University of Natural Resources and Life Sciences Vienna, Muthgasse 18, Vienna, Austria; Faculty of Biochemistry, Poland

## Abstract

Extracorporeal shockwave treatment was shown to improve orthopaedic diseases and wound healing and to stimulate lymphangiogenesis *in vivo*. The aim of this study was to investigate *in vitro* shockwave treatment (IVSWT) effects on lymphatic endothelial cell (LEC) behavior and lymphangiogenesis. We analyzed migration, proliferation, vascular tube forming capability and marker expression changes of LECs after IVSWT compared with HUVECs. Finally, transcriptome- and miRNA analyses were conducted to gain deeper insight into the IVSWT-induced molecular mechanisms in LECs. The results indicate that IVSWT-mediated proliferation changes of LECs are highly energy flux density-dependent and LEC 2D as well as 3D migration was enhanced through IVSWT. IVSWT suppressed HUVEC 3D migration but enhanced vasculogenesis. Furthermore, we identified podoplanin^high^ and podoplanin^low^ cell subpopulations, whose ratios changed upon IVSWT treatment. Transcriptome- and miRNA analyses on these populations showed differences in genes specific for signaling and vascular tissue. Our findings help to understand the cellular and molecular mechanisms underlying shockwave-induced lymphangiogenesis *in vivo*.

## Introduction

Injuries and surgical interventions often lead to local tissue damage and therefore a loss of sufficient nutrient supply and lymphedema in the wound. During the last decade it became possible to partly replace damaged and necrotic tissue by *in vitro* bio-engineered constructs. The need for prevascularization of these constructs with blood and lymphatic vasculature became prominent since both vascular systems are necessary to provide physiological tissue function and hemostasis in the host [1]–[3]. In addition, an alternative therapeutic approach, extracorporeal shockwave treatment (ESWT) was shown to be an effective therapy for a variety of orthopaedic diseases [4]–[6] and to improve wound healing [7]–[10], as the lack of nutrient supply and waste removal in injured tissues can be ameliorated by it.

The biological effects of shockwaves are mediated by a process called mechanotransduction, which influences cell migration, adhesion, apoptosis and viability [11]. It has been elucidated before that mechanotransduction applied by ESWT improves wound healing by inducing angiogenesis via upregulation of endothelial-specific genes and markers such as CD31 [12], vascular endothelial growth factor (VEGF) and VEGF receptor 2 (VEGFR2) [8], [13], [14]. Furthermore, secondary lymphedema in rats was significantly reduced by shockwave-mediated lymphangiogenesis and upregulating VEGF-C, its receptor VEGFR3 and basic fibroblast growth factor (bFGF) [14], [15]. Other recent studies revealed possible mechanisms of shockwave-induced effects *in vitro* and *in vivo*. It is known that endothelial cell activation via *in vitro* shockwave treatment (IVSWT) is caused by toll-like receptor 3 (TLR-3) involvement [16]. Moreover, the investigation of shockwave-promoted bone formation *in vivo* and proliferation studies with different cell types showed that ESWT increases ERK and p38 activation, which is dependent on adenosine triphosphate (ATP) release [17], [18]. Finally, recent studies suggest a role for post-transcriptional regulation via microRNAs (miRNAs) in mediating effects of mechanic endothelial cell stimulation [19].

Although several *in vivo* studies indicate angiogenic and lymphangiogenic effects of ESWT, the *in vitro* effects on lymphatic endothelial cell (LEC) behaviour regarding migration, proliferation, marker expression and vasculogenesis and the underlying molecular mechanisms remain widely unclear. The aims of the present study were to investigate ESWT effects on the biological properties of LECs and the usability of ESWT in vascular regeneration purposes by conducting several well-established proliferation, viability, migration and vasculogenesis assays. Furthermore, changes of LEC marker expression during IVSWT were analyzed. In addition, using transcriptome- and miRNA analyses we screened for mRNA-miRNA networks that might underlie the observed phenotypic changes. To evaluate if shockwaves have different effects on lymphatic compared to blood vascular endothelial cells *in vitro*, human umbilical vein endothelial cells (HUVECs) were used for comparison.

## Materials and Methods

### Cells

Cells were isolated from healthy donors with authorization of a local ethics committee and informed consent by the donor. LECs were isolated from human foreskins via podoplanin selection and immortalized by stable integration of human telomerase as described elsewhere [20], [21]. For the present study, LECs were used in passages 30 to 50. HUVECs were either purchased (C2519A, Lonza, Basel, Switzerland) or isolated from fresh umbilical cords as described before [22]. In brief, freshly donated umbilical cords were stored in antibiotics-containing phosphate buffer saline (PBS) at 4°C for 3 days following 0.2% collagenase treatment (540 U/ml) for 20 min at 37°C and centrifugation in endothelial growth medium (EGM-2, Lonza, Basel, Switzerland). Green fluorescent protein (GFP) expressing HUVEC were purchased from Olaf pharmaceuticals (Cat. No.: GFP, Worcester, USA). Adipose-derived stem cells (ASCs) were isolated from liposuction material as described before [23]. MG63, an osteosarcoma cell line, were purchased from ATCC (CRL-1427, Manassas, USA). LECs, HUVECs and ASCs were maintained in EGM-2 with 5% fetal calf serum (FCS; GE Healthcare, Chalfont St Giles, UK) on surfaces coated with 2 µg/ml bovine fibronectin (Sigma-Aldrich, St. Louis, USA). ASCs were cultured on non-coated surfaces. The MG63 cell line was cultured in Dulbecco's modified Eagle's medium (DMEM; Sigma-Aldrich, St. Louis, USA) supplemented with 10% FCS.

### Antibodies

All antibodies used were diluted according to the manufacturer's datasheets. Fluorescein isothiocyanate (FITC)-conjugated mouse anti-human CD31 (Cat. No. 555445), FITC-conjugated mouse anti-human vascular endothelial cadherin (VE-Cadherin) (Cat. No. 560411) and phycoerythrin (PE)-conjugated mouse anti-human CD146 (Cat. No. 550315) antibodies were purchased from BD Biosciences (Franklin Lakes, USA) and diluted in a 1∶50 ratio. PE-conjugated mouse anti-human VEGFR2 (Cat. No. 130-093-598) was purchased from Miltenyi Biotec (Bergisch Gladbach, Germany) and diluted 1∶50. Polyclonal rabbit antibodies against human podoplanin (Cat. No. 102-PA40S) and human lymphatic vessel endothelial hyaluronan receptor 1 (LYVE-1) (Cat. No. 102-PA50) (1∶100 dilution), and monoclonal mouse anti-human VEGFR3 (Cat. No. 101-M36) (1∶50 dilution) were used from ReliaTech (Wolfenbüttel, Germany). Several experiments were performed with a rabbit anti-human podoplanin antibody kindly provided by Prof. Dontscho Kerjaschki (Medical University of Vienna, Austria). Secondary Alexa Fluor 488-conjugated goat anti-rabbit (Cat. No. A-11034) and goat anti-mouse IgG antibodies (Cat. No. A11001) were purchased from Life Technologies (Carlsbad, USA) and diluted 1∶500.

### *In vitro* shockwave treatment

Shockwaves were applied with a defocused Dermagold 100 device and an OP155 applicator (MTS Medical, Konstanz, Germany). The cells were either stimulated in T25 cell culture flasks, in 15 ml or in 50 ml tubes (Greiner, Kremsmünster, Austria) in PBS with 10% EGM-2. Cells were submerged in a water bath and stimulated with a frequency of 5 Hz, 200 pulses and energy flux densities ranging from 0.03 to 0.19 mJ/mm^2^ at a constant pressure level of 1 bar as described elsewhere [24].

### Proliferation assay

Proliferation of LECs, HUVECs and MG63 was determined by manual counting. The cells were stimulated in T25 cell culture flasks with 200 pulses, 5 Hz and energy flux densities ranging from 0.03 to 0.19 mJ/mm^2^. After IVSWT, cells were detached with trypsin/ethylenediaminetetraacetic acid (EDTA) (Sigma-Aldrich, St. Louis, USA) and seeded to fibronectin-coated 24 well plates (one well for each day for counting was seeded). After 24, 48 and 72 h, cells were enzymatically detached with trypsin/EDTA and counted.

### 2D migration - wound scratch assay

26 mm×76 mm coverglasses (VWR International, Darmstadt, Germany) were washed with 70% ethanol and UV irradiated for 30 min to ensure sterility of the material. The coverglasses were put to petri dishes and coated fibronectin for 10 min. Fibronectin was aspirated and cells were seeded with a density of 4×10^5^ cells/ml to each coverglass for 2 hours at 37°C. Afterwards, additional 8 ml EGM-2 were added to each petri dish. The cells were incubated until the monolayer became confluent. The medium was then aspirated and the monolayer was scratched with a 1000 µl pipet tip (Greiner, Kremsmünster, Austria). The glasses were applied to PBS filled 50 ml tubes directly after scratching and shockwave treated with 0.07 mJ/mm^2^, 5 Hz and 200 pulses. Three images were taken right after stimulation and after 6 h. The reduction of the cell-free area between 0 and 6 h was quantified with ImageJ (NIH, Maryland, USA).

### 3D migration – Cytodex bead assay in fibrin gels

Cells were seeded on Cytodex-3 microcarrier beads (GE Life Sciences, Chalfont St. Giles, UK) by using approximately 400 cells per bead. The bead/cell suspension was shaken gently every 20 min for 3 h at 37°C to ensure homogenous coating of beads. Confluent attachment of cells to beads was achieved by overnight incubation at 37°C. Fibrin clot components (Baxter, Vienna, Austria) were prepared by warming fibrinogen to room temperature (RT) and diluting thrombin to a concentration of 0.4 U/ml in CaCl_2_. 200 µl clots (2.5 mg/ml final concentration, 0.2 U/ml thrombin) were seeded with 100 beads on 26 mm×76 mm coverglasses. After polymerization, the glasses were stimulated in PBS-filled 50 ml tubes with 0.07 mJ/mm^2^, 5 Hz and 200 pulses. The clots were cultured in petri dishes (Greiner, Kremsmünster, Austria) for 5 days. For quantification of migrated cells, the clots were transferred to round coverslips (15 mm diameter, VWR International, Darmstadt, Germany), fixed with 4% paraformaldehyde (PFA; Sigma-Aldrich, St. Louis, USA) overnight and stained with 1 µg/ml 4',6-diamidino-2-phenylindole (DAPI; Sigma-Aldrich, St. Louis, USA) in PBS/1% BSA for 4 hours. Images were taken on a Leica DMI6000B epifluorescence microscope (Leica, Solms, Germany). Quantification was done with ImageJ.

### Adhesion assay

LECs and HUVECs were seeded to either non-coated or fibronectin-coated T25 flasks, Cytodex-1 (non-coated), fibronectin-coated Cytodex-1 or Cytodex-3 (collagen-coated) microcarrier beads (GE Life Sciences, Chalfont St Giles, GB) on day 0. IVSWT was applied on day 1 in cell culture flasks or, in case of bead stimulation, in 15 ml reaction tubes as described above. 24 h later, cells were enzymatically detached with Accutase (Sigma-Aldrich, St.Louis, USA) from flasks and beads for 7 min at 37°C. The beads were removed from cells by pipetting the cell/bead suspension through a 70 µm cell strainer (BD Falcon, Franklin Lakes, USA). The cells were centrifuged at 100×g for 5 min, counted and reseeded to non-coated 48-well plates for exact 25 min. The wells were washed twice with PBS, fixed with 4% PFA for 15 min at 4°C and nuclei were stained with 1 µg/ml DAPI in PBS/1% BSA for 1 h at 4°C. Images were taken on a Leica DMI6000B epifluorescence microscope and the amount of attached cells was quantified with ImageJ.

### Permeability assay

Permeability changes of LEC and HUVEC monolayers after IVSWT were quantified with a 24-well *in vitro* vascular permeability assay kit (ECM644, Millipore, Darmstadt, Germany) according to the manufacturer's protocol. Briefly, 3×10^5^ cells were seeded to the provided membrane inserts and grown until confluence. The inserts were then stimulated in PBS-filled 50 ml reaction tubes (Greiner, Kremsmünster, Austria) with 0.07 mJ/mm^2^, 200 pulses and 5 Hz. FITC-dextran solution was added for 30 min and the flow-through of dextran was measured with a Glomax Multi + detection system (Promega, Madison, USA) before, 0.5 h, 4 h, and 20 h after shockwave treatment.

### Vascular network formation in fibrin gel co-cultures with ASC

Endothelial cell (EC)/ASC co-cultures in fibrin clots were performed as described [25], [26]. Briefly, fibrin gel components (Baxter, Vienna, Austria) were prepared and mixed to 200 µl clots (2.5 mg/ml final concentration, 0.2 U/ml thrombin) together with cells (1×10^5^ EC and 1×10^5^ ASC per clot). The clots were prepared on 26 mm×76 mm coverglasses (VWR International, Darmstadt, Germany) and incubated for 30 min at 37°C. Polymerized fibrin constructs were incubated in EGM-2 and stimulated with 0.07 mJ/mm^2^, 5 Hz and 200 pulses right after polymerization, 2 days and 5 days after preparation. The clots were fixed with 4% PFA overnight on ice and stained with FITC-labelled anti-CD31 antibody (BD Biosciences, Franklin Lakes, USA). Images were taken on a Leica DMI6000B epifluorescence microscope. Tube formation was quantified with Adobe Photoshop CS5 (Adobe Systems, San José, USA) and AngioSys software (TCS Cellworks, London, UK) as described elsewhere [25].

### Flow cytometry analyses

The cells were stimulated on fibronectin-coated T25 flasks. 24 h later, cells were detached from flasks by medium aspiration, washing with PBS and addition of 1 ml prewarmed (37°C) Accutase solution (Sigma-Aldrich, St. Louis, USA) for 7 min at 37°C and cells were centrifuged at 100×g for 5 min. The supernatant was aspirated and cells were resuspended in 1× PBS/1% BSA (Sigma-Aldrich, St.Louis, USA). The cell suspensions were pipetted to polypropylene tubes (BD Falcon, Franklin Lakes, USA) and incubated with antibodies for 30 min on ice. Binding of antibodies against podoplanin, LYVE-1 and VEGFR3 was visualized with an Alexa 488-labeled goat anti-rabbit secondary antibody. The cells were washed twice with PBS and centrifugation steps at 100×g for 5 min. The measurement was performed with a BD FACSCanto II device (BD Biosciences, Franklin Lakes, USA) and data were analyzed using FlowJo software (Tree Star, Ashland, USA).

### Cell sorting and total RNA isolation

LECs were cultivated to a total number of around 7×10^6^ cells. The cells were enzymatically detached, centrifuged at 100×g for 5 min and resuspended in cold EGM-2 to a concentration of 10×10^6^ cells/700 µl. The mixed population was sorted with a MoFlo Astrios cell sorter (BD, Franklin Lakes, USA) according to the forward scatter (FSC) values. The cell suspensions were then centrifuged again at 100×g for 5 min and the medium supernatant was removed. The cells were resuspended in Trizol (Life Technologies, Carlsbad, USA) and chloroform (Carl Roth, Karlsruhe, Germany) was added. The suspension was mixed gently, left resting for 5 min at RT and afterwards centrifuged at 12,000×g for 15 min at 4°C. The RNA was precipitated by isopropanol for 10 min at RT. After centrifugation at 12,000×g for 15 min at 4°C, the RNA pellet was washed with 70% ethanol, dried at RT and resuspended in sterile water. Total RNA quality was estimated from 28S and 18S ribosomal RNA peaks on a Bioanalyzer 2100 instrument using the RNA 6000 Nano Kit (Agilent Technologies, Santa Clara, CA).

### Transcriptome analysis

Consequently, isolated RNA from 3 technical replicates was used to produce biotinylated cRNA using the GeneChip HT 3' IVT Express Kit, then purified and fragmented cRNA was hybridized to GeneChip Human Genome U133 Plus 2.0 arrays (Affymetrix, SC, CA) following the manufacturer's recommendations. The Affymetrix GeneChip Fluidics Station 450 was used to wash and stain the arrays with streptavidin-phycoerythrin according to the standard protocol for eukaryotic targets (IHC kit, Affymetrix). Arrays were scanned with an Affymetrix GeneChip Scanner 3000. The resulting.CEL files were analyzed with Carmaweb (https://carmaweb.genome.tugraz.at/carma/). Briefly, the raw data files were normalized using the RMA method (robust multi– array average). To screen for differentially expressed genes, a moderated t-test (limma) was performed on the normalized datasets, restricted to the 40% of the probesets with the biggest variance over all samples. To exclude normalization specific artifacts, another normalization method, MAS5, values scaled to 200, was applied to the .CEL files, and differentially expressed genes were again determined by the moderated t-test (limma) on the normalized datasets, restricted to the 40% of the probesets with the biggest variance over all samples. Depending on these two normalization methods, two datasets for the 100 best candidates (100 lowest p-values) were generated, and further combined and analyzed in Microsoft Excel.Array data were submitted to Gene Expression Omnibus (GEO) and are available under the Accession Number GSE62510.

### MicroRNA microarray hybridization

miRNA microarray experiments were run as described previously [27]. In brief, epoxy-coated Nexterion glass slides were spotted using the miRBase version 16.0 locked nucleic acid (LNA) probe set consisting of 2,367 probes against human, mouse and rat miRNAs in 8 replicates (Exiqon, Denmark). For hybridization 1000 ng total RNA extracts from two biological replicates of each sorted cell population were used. End-labeling of miRNAs with Cy3 was performed using the Exiqon Power Labeling Kit (Exiqon, Denmark) together with synthetic spike-in controls according to the instructions of the manufacturer. Slides were hybridized after an initial wash step with Cy3-labeled total RNA samples for 16 h at 56°C in a Tecan HS 400 hybridization station at high agitation speed (Tecan, Austria). After hybridization, automated washing, and slide drying, arrays were immediately scanned using the Roche Nimblegen MS200 scanner (Roche, Germany) at 10 µM resolution, 100% laser-power and auto-gain settings.

### MiRNA microarray data analysis

Feature extraction from high-resolution tiff-images was performed using GenePix software (Molecular Devices, Sunnyvale, CA). Background correction and between-array normalization were performed with LIMMA under R/Bioconductor [28] using the *minimum* method and *quantile* normalization, respectively. Log_2_-transformed fold changes of miRNAs in the podoplanin^low^ and podoplanin^high^ subpopulations were calculated using *lmfit*. Due to the exploratory character of this study, fold changes were prioritized against p-values, and therefore miRNAs were ranked according to log_2_ fold change between podoplanin^high^ versus podoplanin^low^ subpopulations and the top50 candidates' ±1.5 fold changes were considered as potentially differentially expressed.

Putative interactions between regulated miRNAs and mRNAs were investigated using miRWalk [29]: the top up- and down-regulated genes were submitted for target prediction considering 9 different algorithms (DIANAmT, miRanda, miRDB, miRWalk, RNAHybrid, PICTAR5, PITA, RNA22, Targetscan). Only miRNA∼mRNA interactions predicted by ≥50% of algorithms were retained, and evaluated for negatively correlating expression values (log_2_ fold change +/− 0.5) in the generated mRNA and miRNA datasets.

### MiRNA qPCR analysis

In order to confirm microarray data, miRNA RT-qPCR analysis was performed. A total of 10 ng RNA per sample was reverse transcribed using the Universal cDNA synthesis II Kit in 10 µl reactions (Exiqon, Denmark). Subsequently, cDNA samples were diluted 1∶40 and for each reaction, 4 µl of diluted cDNA were mixed with 1 µl of combined forward and reverse primer assays and 5 µl SYBR Green Mastermix (Exiqon, Denmark). All qPCR reactions were performed after initial denaturation at 95°C (10 min) for 45 cycles in triplicates on a Light Cycler 480 II using a two-step protocol with 60°C elongation (60 s) and 95°C denaturation (10 s). For the analysis Cp values were calculated using the second derivative maximum method and used for relative quantification using delta-delta-Ct analysis. 5S rRNA and RNU6 were used as internal reference genes.

### Statistics

Statistical differences were calculated using Student's *t* test when comparing 2 groups. The comparison of 3 or more groups was assessed by 1-way ANOVA with Tukey-Kramer post testing. All data sets are presented as mean +/− standard deviation unless otherwise noted. P values less than 0.05 were considered as significant. All statistical analyses were performed with GraphPad Prism 4.0 software (GraphPad, San Diego, USA).

## Results

### IVSWT influences LEC migration, proliferation, adhesion and permeability

**Figure 1:**
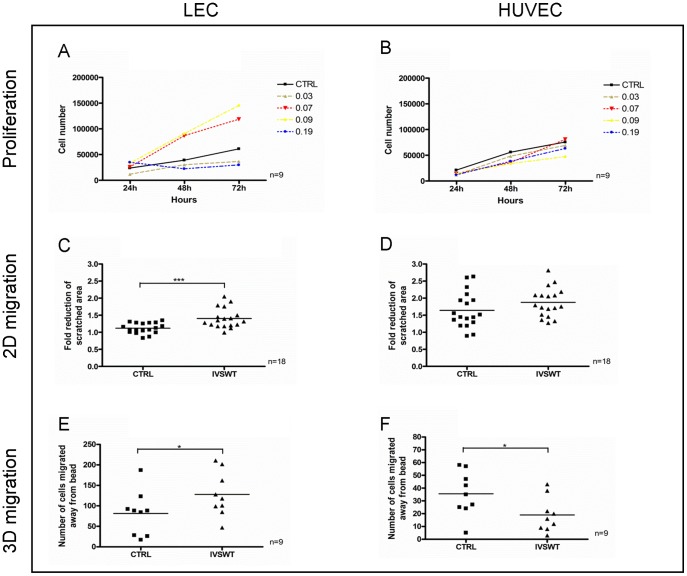
LEC and HUVEC proliferation and migration changes upon IVSWT. (A) LEC proliferation was enhanced by stimulation with 0.07 and 0.09 mJ/mm^2^, but decreased by 0.03 and 0.19 mJ/mm^2^. (B) HUVEC proliferation was unaffected by IVSWT. (C) The reduction of a scratched, cell-free area within a time frame of 6 h was significantly enhanced by IVSWT in LECs. (D) No changes of migration upon IVSWT were observed in HUVECs. (E) IVSWT mediated a significant migration of LECs away from Cytodex-3 microcarrier beads embedded in fibrin gels. (F) HUVEC 3D migration was reduced in a 3D migration model. P-values: *** ≤0.01, ** ≤0.1, * ≤0.5.

Recent *in vivo* studies revealed ESWT-mediated upregulation of VEGF and its receptor VEGFR2 [30], [31] as well as VEGF-C and VEGFR3 [14], [15]. To reproduce these findings *in vitro*, LECs and HUVECs were stimulated with different energy flux densities at constant pulse number and frequency to ascertain possible energy-dependent proliferation changes. As it is shown in Fig. 1A, proliferation of LECs was enhanced when cells were stimulated with 0.07 and 0.09 mJ/mm^2^. In contrast, 0.03 and 0.19 mJ/mm^2^ suppressed proliferation compared to a non-stimulated control group. However, HUVEC proliferation was not altered by IVSWT using the same parameters used to determine LEC proliferation changes (Fig. 1B). We further examined IVSWT effects on the non-endothelial cell line MG63 and found no changes in proliferation after treatment (S1 Figure in S1 File). To measure the influence of shockwaves on cell migration artificially created scratches in cell monolayers were stimulated with 0.07 mJ/mm^2^ since different preliminary experiments identified this energy flux density level as the one inducing the highest responses of LECs (data not shown). The change of migration of LECs after 6 h in a 2D set-up was significantly higher with IVSWT compared to a non-stimulated control (Fig. 1C) whereas HUVEC migration remained unchanged after stimulation (Fig. 1D). Moreover, LEC and HUVEC migration responses to IVSWT were further determined by using a more physiological 3D migration model. Fig. 1E demonstrates a significant increase of LEC migration in a 3D setup upon IVSWT. HUVEC migration was significantly decreased after treatment (Fig. 1F). LEC adhesion assays revealed a significant reduction when cells were stimulated on Cytodex-1 microcarrier beads, however no effects were observed when cells were stimulated on other extracellular matrices (S2A Figure in S1 File). In addition, HUVEC adhesion did not change at all (S2B Figure in S1 File). Moreover, the permeability of LEC monolayers was higher 30 minutes after IVSWT compared to non-treated cells, but no differences were observable after 20 hours (S2C Figure in S1 File).

### IVSWT significantly enhances in vitro vasculogenesis

**Figure 2:**
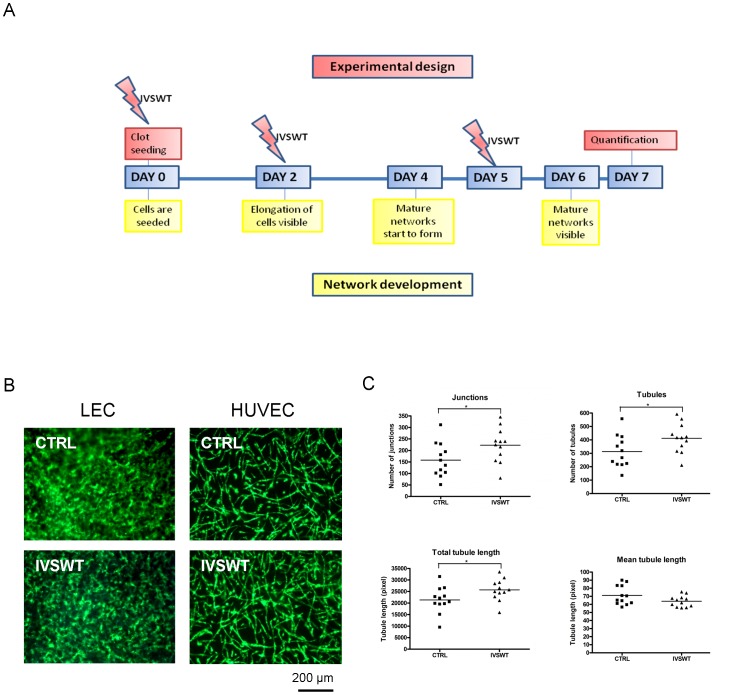
IVSWT-mediated HUVEC vasculogenesis. (A) Overview of the treatment setup for stimulation of EC/ASC co-cultures in fibrin. (B) Fluorescent images of non-treated versus treated LEC and HUVEC networks on day 7. (C) Quantifications of HUVEC networks. IVSWT increased the number of junctions, tubules and the total tubule length. The mean tubule length was decreased. P-values: *** ≤0.01, ** ≤0.1, * ≤0.5.

We have shown before that endothelial cells form vascular networks in fibrin gels in the presence of ASCs after one week [25]. In the present study, this model was used to determine the ability of IVSWT to induce vascular tube formation *in vitro*. Various shockwave treatment setups were tested (S3A–S3C Figures in S1 File). Finally, a three times treatment turned out to be necessary for visualizing the influence of shockwaves. Fig. 2A illustrates the time schedule for shockwave treatment of the gels. The gels were stimulated right after preparation, and on the second and fifth day. Applying these parameters IVSWT enhanced HUVEC-mediated vasculogenesis *in vitro* (Fig. 2B). The total number of junctions, tubules and the total tubule length were significantly higher in the stimulated group compared to non-treated controls whereas the mean tubule length decreased (Fig. 2C). In contrast, using LECs instead of HUVECs, we did not find significant differences between shockwave-treated and control groups (Fig. 2B).

### IVSWT induces an upregulation of podoplanin in LECs

**Figure 3:**
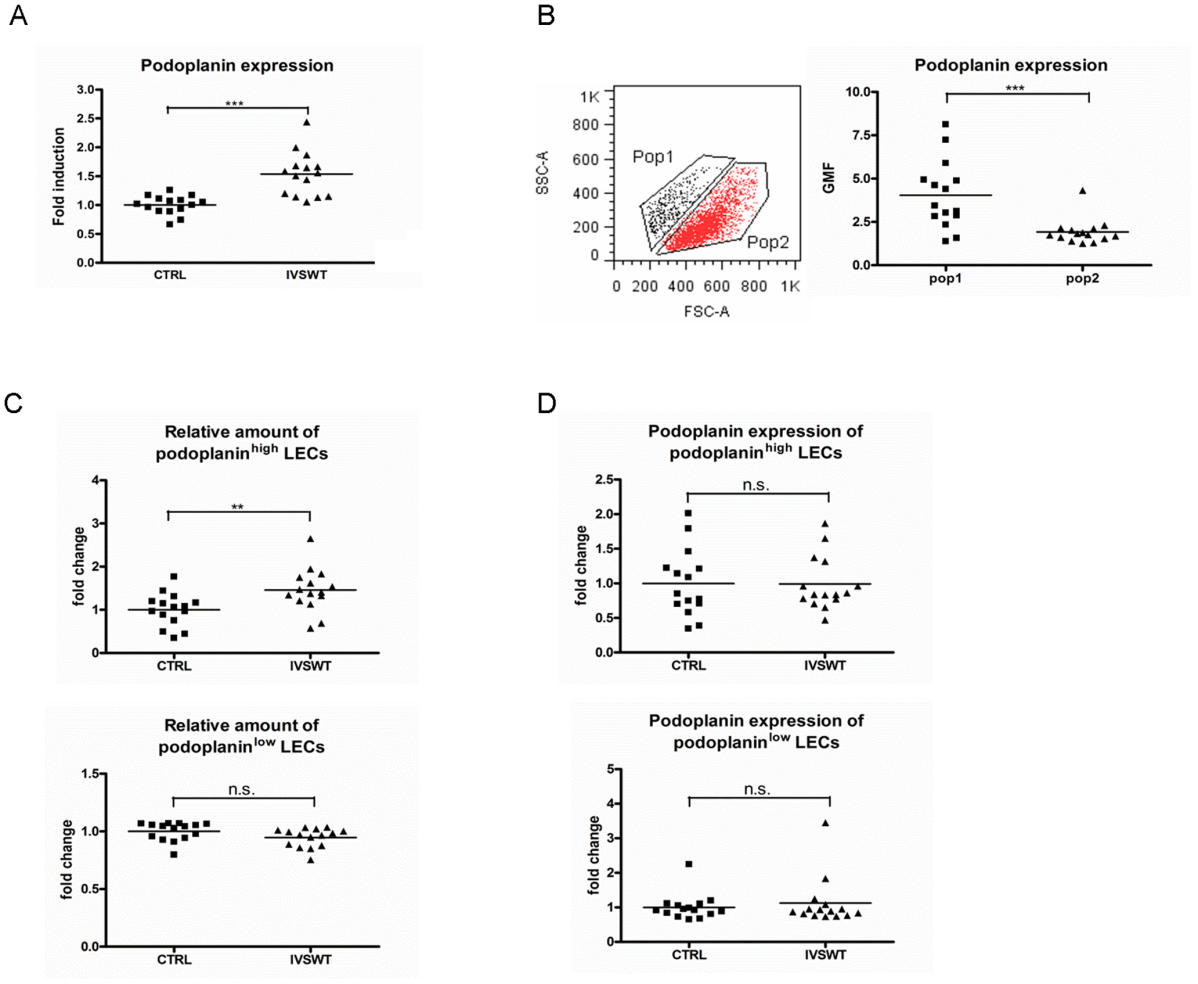
Flow cytometry analyses of IVSW-treated LECs. (A) Podoplanin expression on LECs was significantly enhanced by IVSWT. (B) LEC populations differ in FSC values and podoplanin expression. (C) Shockwave stimulation mediates a morphology change of LECs by increasing the amount of podoplanin^high^ LECs. The amount of podoplanin^low^ LECs did not change significantly. (D)Neither the podoplanin expression on podoplanin^high^, nor the expression on podoplanin^low^ LECs changed upon IVSWT. All analyses were performed with n = 15. P-values: *** ≤0.01, ** ≤0.1, * ≤0.5.

To identify possible target molecules involved in IVSWT influences on LEC behavior, flow cytometry analyses were performed. Lymphatic endothelial markers VEGFR3 and LYVE-1 as well as pan-endothelial markers such as CD31, VEGFR2 and CD144 showed no regulation upon IVSWT in LEC and HUVEC (S4A,B Figure in S1 File). In contrast, the expression of podoplanin was significantly increased after IVSWT in LECs (Fig. 3A). This effect was highly energy flux density dependent (S4C Figure in S1 File). Whereas low (0.03 mJ/mm^2^) and high (0.19 mJ/mm^2^) energy flux densities downregulated podoplanin expression, increases were observed at average energy flux densities (0.07 and 0.09 mJ/mm^2^), the same energy flux densities that also increased proliferation. Interestingly, we continuously identified two different subpopulations of LECs differing in their forward scatter (FSC) values (Fig. 3B, left panel). By gating these populations we found that the smaller cells expressed more podoplanin (mean geometric mean 4.04) than the larger population (mean geometric mean 1.92, Fig. 3B, right panel). The populations are further termed podoplanin^high^ and podoplanin^low^ LECs throughout the manuscript. IVSWT mediated a significant increase in the relative amount of podoplanin^high^ LECs. The podoplanin^low^ LEC population concomitantly decreased, albeit not to a significant extent (Fig. 3C). However, IVSWT did not alter the podoplanin expressionin the respective populations (Fig. 3D). Thus, IVSWT did not upregulate podoplanin *per se*, but mediated an increase in the podoplanin^high^ LEC population.

In order to gain insight into differences in global gene expression we sorted the podoplanin^high^ and podoplanin^low^ populations by flow cytometry and performed microarray analysis on total RNA isolated from these populations. The 100 most differentially expressed transcripts of the two populations were selected using two different normalization methods as detailed in materials and methods. When screened for maximal differential gene expression in the podoplanin^high^ and podoplanin^low^ populations, the two normalization methods resulted in different top candidate lists. Only 12 transcripts of the 100 transcripts per list were commonly found in both lists. Of the best 20 (lowest p-values) RMA-normalized genes, only 5 (25%) were also found in the 100 most regulated mas5 normalized genes, while only 1 (5%) gene of the best 20 mas5 normalized genes was found amongst the 100 most regulated RMA normalized genes. These differences raised concerns about selecting high numbers of false positive candidates by both normalization methods. To rule out this potential high number of false positive differentially regulated genes, the two lists with the 100 most differentially regulated genes were combined, and an average of meanM (log2 transformed fold difference) and the respective statistic analyses (raw p-values, Bonferroni adjusted p-value (strong control of the family wise error rate), BH (Benjamini and Hochberg – strong control of the false discovery rate) was calculated. To select the most differentially regulated genes, the criteria for the means from both datasets were a raw p-value of <0.05, a BH value of <0.5, and a Bonferri of <1 and only genes more than 2-fold regulated were selected (average meanM >1 AND <−1). From the remaining 40 genes, 10 found to be inversely regulated when comparing the different normalization methods were excluded as false positives, and additionally 5 internal Affymetrix probe-sets were excluded from the final list. The more-than-2-fold differentially regulated genes found in both top 100 lists are Ras and EF-hand domain-containing protein (RASEF), Nuclear Enriched Abundant Transcript 1 (NEAT1), radiation-sensitive mutant 21 (RAD21), glutathione peroxidase 3 (GPX3), matrix Gla protein (MGP), flavin-containing monooxygenase 3 (FMO3), serine protease inhibitor E2 (SERPINE2) and AF194537.

**Figure 4:**
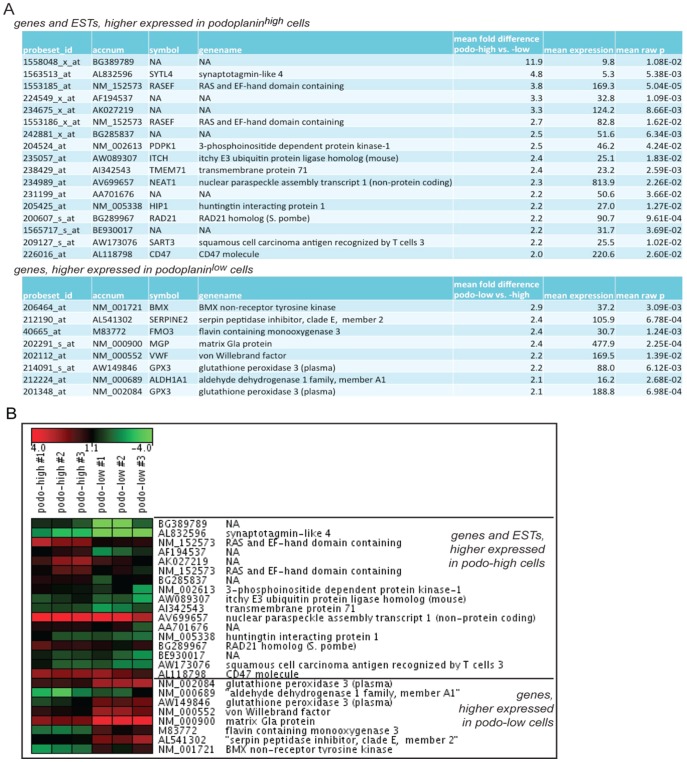
Gene expression profile of sorted subpopulations. (A) Transcripts with more than two-fold stronger expression in one of the respective populations (B) Heatmap visualization of 3 replicates showing log_2_-transformed gene expression of podoplanin high and podoplanin low LECs. Affymetrix.CEL files were mas5 normalized. Log2-transformed expression values were normalized centred to the median of the 25 plotted transcripts.

The final list with 25 of the more than two-fold differentially regulated transcripts (Fig. 4A) was sorted by mean fold expression and the log2 transformed expression values from the mas5 normalized list were plotted as a heatmap using GenesisWeb (https://carmaweb.genome.tugraz.at/genesis/). As normalization method “Median Center Experiments” was applied (Fig. 4B).

Gene ontology (GO) analysis showed that mainly genes involved in cell signaling and genes involved in functions of membrane or cytoplasmatic vesicles are differently expressed (S5A Figure in S1 File). Furthermore, after extending the GO analysis to tissue expression, 6 out of 7 annotated genes higher expressed in the podoplanin^high^ population are expressed in normal connective tissue, 5 in normal vascular tissue such as von Willebrand factor (vWF), non-receptor tyrosine kinase BMX, matrix Gla protein (MGP), SERPINE2 and aldehyde dehydrogenase H1A1 (ALDH1A1). Genes higher in the podoplanin^high^ population are expressed in bone marrow, in acute myelogenous leukemia and in stem progenitor cells CD34+ and CD38+ (S5B Figure in S1 File).

**Figure 5:**
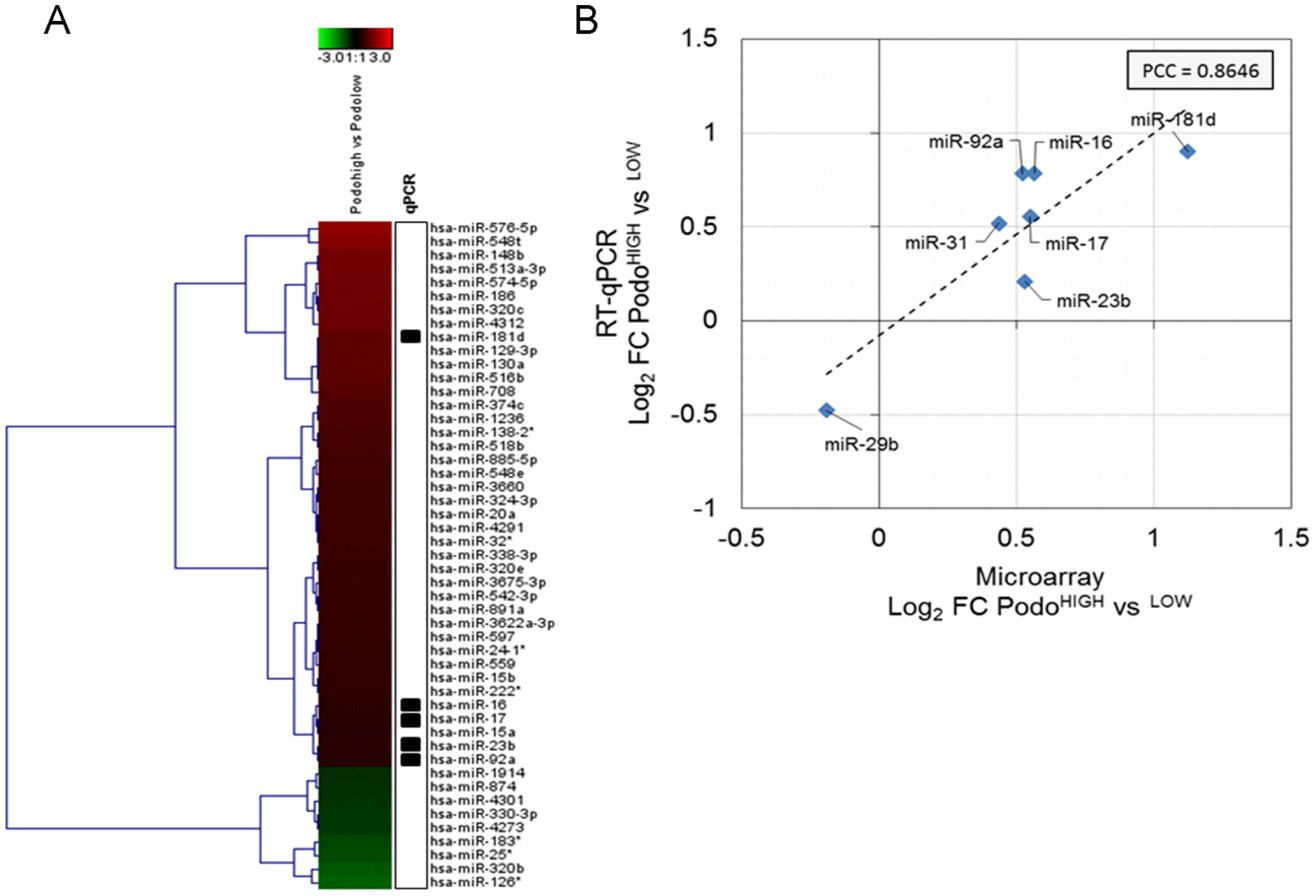
Regulation of miRNA transcription in sorted subpopulations. (A) Heatmap visualization of log_2_-transformed fold changes between podoplanin^high^ and podoplanin^low^ LECs. The top 50 regulated miRNAs according to log_2_-fold change were chosen for visualization. Average log_2_ fold changes from n = 2 biological replicates per sample are shown. (B) Specific miRNAs were selected for confirmation of fold-changes by quantitative PCR (indicated by boxes in the heatmap). Average log_2_-transformed fold changes between podoplanin^high^ and podoplanin^low^ LECs derived from microarray and RT-qPCR (reference gene  = 5S rRNA) are shown (n = 2 per sample) and linear correlation was estimated using Pearson correlation (PCC  = 0.8646).

Moreover, we also analyzed the miRNome from the podoplanin^high^ and podoplanin^low^ populations. Hybridization shows that after imposing a fold change cut-off >1.5 fold, the majority (41 miRNAs) were upregulated in podoplanin^high^ LECs, compared to 9 down-regulated miRNAs (Fig. 5A). To test the validity of these results, the expression of the top-regulated miRNAs (miR-16, miR-17, miR-181d, miR-23b, miR-92a) as well as two other miRNAs (miR-29b, miR-31) were confirmed by qPCR analysis, resulting in a significant correlation as indicated by a pearson correlation coefficient of 0.86 (Fig. 5B).

Interestingly, we found miR29-b differentially regulated. This miRNA has been reported to regulate the expression of podoplanin by direct targeting the 3′ untranslated region of the podoplanin transcript [32], which, however does not differ between the two populations on the mRNA level.

A more thorough analysis of mRNA∼microRNA correlation analysis showed that 5 out of the top 8 transcripts, which were lower expressed in the podoplanin^high^ population, exhibited putative miRNA regulation networks (Table S1 in File Supplementary Information). For example, BMX and vWF were found to share regulation by the miR-15a/b-16 cluster, which was found to be significantly up-regulated in podoplanin^high^ LECs (S6 Figure in S1 File). *Vice versa*, 6 out of 12 transcripts up-regulated in podoplanin^high^ LECs were found to harbour miRNA binding sites for down-regulated miRNAs (Table S2 in File Supplementary Information). These data point towards epigenetic effects of IVSWT on lymphatic endothelial cells.

## Discussion

Based on several published *in vivo* studies which indicate pro-(lymph)angiogenic effects of ESWT, the present study was designed to employ established methods to analyse shockwave-induced lymphangiogenesis *in vitro*. We show that shockwaves alter the biological properties of LEC in terms of proliferation, migration, morphology, marker profiles and gene expression.

Kubo et al. (2010) reported enhanced levels of VEGF-C and VEGFR3 in rat tissues after ESWT, indicating a positive effect on LEC proliferation *in vivo* [15]. We confirmed these data *in vitro* by demonstrating an increase of LEC proliferation after treatment with average energy flux densities 0.07 and 0.09 mJ/mm^2^. However, the positive effects of IVSWT on proliferation were strongly energy flux density dependent as low and high levels (0.03 and 0.19 mJ/mm^2^) decreased the proliferation rate of LECs. Weihs et al. (2014) showed that varying energy flux density levels result in different proliferation and signaling responses [18]. Mittermayr et al. (2012) and Kuo et al. (2009) highlighted the importance of parameter influences on the outcomes of ESWT studies [8], [33]. Different energy flux densities, frequencies, pulse numbers, cell monolayer confluencies, and distances to the shockwave transducer during stimulation were performed by proliferation and flow cytometry experiments to identify the most effective parameters for IVSWT of LECs. Optimal treatment parameters were elaborated as: 5 cm distance to the transducer with 0.07 mJ/mm^2^, 5 Hz, and 200 pulses. It has to be noted, however, that these parameters are specific for LECs, since HUVECs responded differently, thus underlining the necessity of testing SW parameters for every cell type before experimentation.

The differing response of LECs and HUVECs to distinct stimulation was observed not only in proliferation assays, but also in 2D and 3D migration experiments. Whereas LECs showed a significantly higher migration in wound scratch assays, HUVEC migration was not altered by IVSWT. To determine the migration responses after shockwave stimulation in a more physiological environment, 3D migration assays were performed. Interestingly, LEC migration away from confluently coated Cytodex-3 beads was enhanced after stimulation whereas HUVEC migration was decreased. This converse effect may originate from an enhancement of adhesion molecules present on HUVECs but not on LECs. In addition, results from adhesion experiments demonstrate that LEC reattachment ability is only significantly altered when cells were seeded to Cytodex-1 beads. However, HUVEC reattachment remained stable after treatment in all observed conditions indicating a strong influence of extracellular matrix on adhesion during stimulation since beads were not embedded into fibrin gels for the adhesion assays in contrast to the 3D migration experiments.

Several *in vivo* studies showed an IVSWT-mediated enhancement of angiogenesis and lymphangiogenesis [12]–[15], [30], [31]. Therefore, we decided to analyze vasculogenic effects in our previously developed co-culture model of ECs with ASCs [25], [26]. It was published before that outgrowth endothelial cells (OECs) form vascular networks in presence of ASCs in fibrin [25]. In contrast to blood vessel networks, lymphatic vasculature consists of blind-ended capillaries [34]. In the current study, the formation of blind-ended LEC structures was also observed. Moreover, mature, quantifiable HUVEC networks were visible after one week in HUVEC/ASC co-cultures. First of all, different treatment time points and quantities were tried to identify optimal stimulation. It was shown before by Davis et al. (2009) that a single treatment with shockwaves has no adverse effect of burn wound closure in mice and several treatments were necessary to suppress proinflammatory immune response [35]. No effect was observed when co-cultures were stimulated on the first day and quantified after one week, confirming the results of Davis et al. (2009) by demonstrating that consecutive treatments are necessary to observe effects in long-term experiments *in vitro* and *in vivo*. Cells start to elongate on the second day after seeding and the maturation of the formed networks takes place between day 5 and 7. The most effective treatment was reached when the cells were stimulated on the first day, and on the crucial time points day 2 and on day 5 after seeding. While the amount of junctions, tubules and the total length of all tubules significantly increased via IVSWT, the mean length decreased indicating that a much more interconnected network was present in the stimulated group. Surprisingly, experiments with an EC∶ASC ratio of 1∶0.5 instead of 1∶1 revealed a suppressing effect of IVSWT on network formation. Furthermore, IVSWT showed no influence in fibrin clots with HUVEC mono-cultures. We speculate that this reduction of vasculogenic potential is connected to the decreased 3D migration of HUVECs in fibrin gels. Presumably, the presence of ASCs reverses the suppressive effect of IVSWT on HUVEC movement in the clot.

We further evaluated endothelial surface marker expression upon shockwave treatment. Although it was published before that HUVEC marker expression changed via IVSWT [16], we could not confirm these data in our study. This may be due to cell donor variability or shockwave device specialities. Our results further demonstrated that podoplanin, a LEC-specific marker [36]–[38], was significantly upregulated after IVSWT. Since two cell populations differing in size and podoplanin expression became visible during the analyses, we investigated these different sub-groups in more detail. Interestingly, IVSWT mediated a morphology change which led to a visible shift from the podoplanin^low^ larger cells to the podoplanin^high^ smaller population. However, both populations had the same induction of podoplanin after IVSWT, indicating that the total increase of podoplanin is merely based on the population shift and increase in the amount of podoplanin^high^ cells. Further marker expression analyses revealed that other pan-endothelial and lymphatic specific markers on LECs are unaffected by IVSWT indicating the IVSWT act on distinct cell functions and does not show an universal increase of markers or cell functions in contrast to published data [12], [13], [15], [16]. Moreover, the importance of choosing the optimal stimulation parameters mentioned before was enforced by showing a strong energy flux density dependence of podoplanin upregulation in LECs and cell type specificity since HUVECs showed different responses than LECs.

The IVSWT-promoted population shift of LECs revealed several differences in the transcriptome. By comparing both subpopulations of LECs we found several genes up- and downregulated. Interestingly, there was no difference in the expression of podoplanin on the mRNA level, suggesting posttranscriptional or –translational regulation by shockwaves. VWF expression differed comparing the podoplanin^high^ with the podoplanin^low^ populations. It has been shown before that shear stress mediates an upregulation of vWF in late endothelial progenitor cells (EPCs) [39] suggesting that IVSWT may have similar effects on LECs. This could be explained by concomitant down-regulation of several miRNAs, which are predicted to target vWF. Among these, the miR-15a/b-16 cluster is known to negatively regulate cell proliferation and to play a role in lymphocytic leukemia [40]. The downregulation of both, the endothelial non-receptor kinase BMX and vWF in podoplanin^high^ cells could be explained by the fact that an increased BMX expression leads to an enhanced vascular response (vWF expression) in mice [41] and both transcripts share the regulation via the miR-15a/b-16 cluster. We were able to identify the expression of several other miRNAs regulating transcripts which were found to be up- or downregulated in podoplanin^high^ versus podoplanin^low^ LECs indicating regulation on the posttranscriptional level of cells treated with IVSWT. SERPINE2, shown to be upregulated in hemangioma derived endothelial cells [42] and mediating lymph node metastasis in testicular cancer [43], was downregulated in the podoplanin^high^ population most likely by an interaction with miR-513a-3p, miR-186 and miR-559. The enhanced 2D migration of LECs after IVSWT could be explained by an upregulation of the 3-phosphoinositide dependent protein kinase-1 (PDPK1) transcript by miR-874 and miR-330-3p, since it has been shown that PDPK1 promotes cell chemotaxis [44]. CD47 was shown to be a downstream target for Ets-1, a transcription factor expressed by endothelial cells during angiogenesis [45]. Since proliferation and migration are prerequisites for angiogenesis as well as for lymphangiogenesis we suggest that the enhancing effect of IVSWT on LEC proliferation and migration could be at least partly mediated by an increased CD47 expression. Moreover, enhanced RAD21 expression, regulated by miR-320b as is CD47, may contribute to increased proliferation [46]. Direct comparisons of the two populations for genes which might be involved in mechanosensing (e.g. the Duffy blood group chemokine receptor DARC or transient receptor potential cation channels) or tip/stalk-cell determination (genes of the notch- and delta-like families) did not reveal more than 1.3 fold differences. Holfeld et al. (2014) showed that TLR-3 was upregulated by IVSWT in HUVECs [16]. However, we could not confirm this finding in LECs, again indicating a strong cell-type dependent influence of IVSWT on the transcriptome. Interestingly, podoplanin^high^ and podoplanin^low^ LECs have been described in the skin *in vivo* [47]. These different cell types constitute lymphatic capillaries (podoplanin^high^) and lymphatic precollector vessels (podoplanin^low^). According to the gene expression changes of LECs after IVSWT shockwaves might lead to a conversion from LECs forming precollector vessels to capillary-forming cells with higher lymphangiogenic potential, thus providing an explanation for the reported *in vivo* effects of shockwaves on lymphatic vasculature. Taken together, our transcriptome and miRNome analyses reveal differences in signalling and endothelial-specific genes involved in vascular tube formation and remodelling. Since these biological processes are vital to wound healing and tissue repair, shockwave-induced cellular events might help to explain the beneficial effects of ESWT on injured tissues.

One limitation of this study is the use of immortalized LECs which are not suitable for clinical use. However, we and others have shown that LECs immortalized by human telomerase retain their biological properties as well as endothelial marker expression [48]–[51], thus justifying their use for this study.

Extracorporeal shockwave therapy is an emerging treatment strategy for a variety of disorders. The plethora of data pointing to improved angiogenesis and lymphangiogenesis led us to investigate how shockwaves influence lymphatic endothelial cells on a molecular level. We show data indicating that IVSWT does change cell morphology which further leads to gene regulation and miRNA expression changes. More studies aiming to elucidate molecular mechanisms of signal transduction within these cell types will shed further light on the complex processes induced by shockwaves.

## Supporting Information

S1 File **Supplementary Information. S1 Figure, Influences of IVSWT on MG63 proliferation.** Shockwave treatment with different energy flux densities had no visible effects on MG63 proliferation. **S2 Figure, IVSWT-induced changes of EC adhesion and LEC permeability.** (A) The ability of LECs to reattach to a fibronectin-coated surface after shockwave treatment was significantly decreased when cells were stimulated on Cytodex-1 microcarrier beads. (B) HUVEC adhesion was not influenced by IVSWT. (C) Quantification of LEC monolayer permeability demonstrates an increase in permeability right after treatment which decays after 4 hours. P-values: *** ≤0.01, ** ≤0.1, * ≤0.5. **S3 Figure, IVSWT-mediated influences on** ***in vitro*** **vasculogenesis.** (A) Stimulation of HUVEC/ASC co-cultures on day 0 with following incubation for 7 days showed no visible changes in the amount of junctions, tubules, total and mean length of the developed networks. (B) Network stimulation on day 2 after seeding with fixation on day 4 revealed no effect of IVSWT on vasculogenesis. (C) Choosing an EC∶ASC ratio of 1∶0.5 instead of 1∶1 revealed a converse effect to the 1∶1 ratio results (Shown in figure 2). The amount of junctions, tubules and the total length of tubules decreased whereas the mean tubule length increased. Scale bar  = 200 µm. P-values: *** ≤0.01, ** ≤0.1, * ≤0.5. **S4 Figure, Flow cytometry analyses of LEC and HUVEC marker expression after IVSWT.** (A) Cell surface expression of CD31, VE-Cadherin, VEGFR2, VEGFR3 and LYVE-1 on LECs did not change upon IVSWT whereas a significant upregulation of podoplanin was observed. (B) The expression of CD31, VE-Cadherin, CD146, VEGFR2 and Tie-2 on HUVECs did not change significantly after IVSWT. (C) The upregulation of podoplanin on LECs is energy flux density dependent. 0.03 and 0.09 mJ/mm^2^ suppressed, whereas 0.07 and 0.09 mJ/mm^2^ increased podoplanin expression. P-values: *** ≤0.01, ** ≤0.1, * ≤0.5. **S5 Figure, Gene ontology (GO) Analysis of genes differentially expressed in the sorted LEC subpopulations.** (A) Functional annotation cluster of all annotated genes that are higher expressed in podoplanin^high^ cells (A1), or higher expressed in podplanin^low^ cells (A2). (B) Functional annotation chart, including tissue expression, of all annotated genes that are higher expressed in podoplanin^high^ cells (B1), or higher expressed in podplanin^low^ LECs (B2). All analyses were performed using DAVID (Database for Annotation, Visualization and Integrated Discovery) at http://david.abcc.ncifcrf.gov/home.jsp. **S6 Figure, Correlation analysis of putative mRNA∼ microRNA networks in IVSWT treated LECs.** (A) Network visualization of mRNA transcripts down-regulated in podoplanin^high^ LECs (yellow boxes) and targeting microRNAs (blue boxes) with negatively correlating expression (i.e. up-regulated in podoplanin^high^ LECs). (B) Analogous network visualization for mRNA transcripts up-regulated in podoplanin^high^ LECs and targeting microRNAs with negatively correlating expression. **S1 Table, Predicted microRNA binding sites and correlation analysis in down-regulated genes (Podoplanin^high^ vs Podoplanin^low^).** 5 genes showed predicted binding sites and correlation with miRNAs. **S2 Table, Predicted microRNA binding sites and correlation analysis in up-regulated genes (Podoplanin^high^ vs Podoplanin^low^).** 9 genes showed predicted binding sites and correlation with miRNAs.
(DOC)

## References

[1] Nomi M, Atala A, Coppi PD, Soker S (2002) Principals of neovascularization for tissue engineering. Molecular Aspects of Medicine 23(6):463–483. 12385748 10.1016/s0098-2997(02)00008-0

[2] Mikos AG, Sarakinos G, Leite SM, Vacant JP, Langer R (1993) Laminated three-dimensional biodegradable foams for use in tissue engineering. Biomaterials 14(5):323–330. 8507774 10.1016/0142-9612(93)90049-8

[3] Soker S, Machado M, Atala A (2000) Systems for therapeutic angiogenesis in tissue engineering. World Journal of Urology 18(1):10–18. 10766038 10.1007/pl00007070

[4] Gerdesmeyer L, Wagenpfeil S, Haake M, Maier M, Loew M, et al (2003) Extracorporeal shock wave therapy for the treatment of chronic calcifying tendonitis of the rotator cuff: a randomized controlled trial. JAMA 290(19):2573–2580. 14625334 10.1001/jama.290.19.2573

[5] Haupt G (1997) Use of extracorporeal shock waves in the treatment of pseudarthrosis, tendinopathy and other orthopedic diseases. The Journal of Urology 158(1):4–11. 9186313 10.1097/00005392-199707000-00003

[6] Wang CJ, Wang FS, Yang KD, Weng LH, Ko JY (2006) Long-term results of extracorporeal shockwave treatment for plantar fasciitis. The American journal of sports medicine 34(4):592–596. 16556754 10.1177/0363546505281811

[7] Kuo YR, Wang CT, Wang FS, Chiang YC, Wang CJ (2009) Extracorporeal shock-wave therapy enhanced wound healing via increasing topical blood perfusion and tissue regeneration in a rat model of STZ-induced diabetes. Wound Repair and Regeneration 17(4):522–530. 19614917 10.1111/j.1524-475X.2009.00504.x

[8] Mittermayr R, Antonic V, Hartinger J, Kaufmann H, Redl H, et al (2012) Extracorporeal shock wave therapy (ESWT) for wound healing: technology, mechanisms, and clinical efficacy. Wound Repair and Regeneration 20(4):456–465. 22642362 10.1111/j.1524-475X.2012.00796.x

[9] Wang CJ, Kuo YR, Wu RW, Liu RT, Hsu CS, et al (2009) Extracorporeal shockwave treatment for chronic diabetic foot ulcers. Journal of Surgical Research 152(1):96–103. 18619622 10.1016/j.jss.2008.01.026

[10] Schaden W, Thiele R, Kölpl C, Pusch M, Nissan A, et al (2007) Shock wave therapy for acute and chronic soft tissue wounds: a feasibility study. Journal of Surgical Research 143(1):1–12. 17904157 10.1016/j.jss.2007.01.009

[11] Jaalouk DE, Lammerding J (2009) Mechanotransduction gone awry. Nature Reviews Molecular Cell Biology 10(1):63–73. 19197333 10.1038/nrm2597 PMC2668954

[12] Zins SR, Amare MF, Tadaki DK, Elster EA, Davis TA (2010) Comparative analysis of angiogenic gene expression in normal and impaired wound healing in diabetic mice: effects of extracorporeal shock wave therapy. Angiogenesis 13(4):293–304. 20848181 10.1007/s10456-010-9186-9

[13] Yip HK, Chang LT, Sun CK, Youssef AA, Sheu JJ, et al (2008) Shock wave therapy applied to rat bone marrow-derived mononuclear cells enhances formation of cells stained positive for CD31 and vascular endothelial growth factor. Circulation journal: Official Journal of the Japanese Circulation Society 72(1):150–156. 18159117 10.1253/circj.72.150

[14] Serizawa F, Ito K, Matsubara M, Sato A, Shimokawa H, et al (2011) Extracorporeal shock wave therapy induces therapeutic lymphangiogenesis in a rat model of secondary lymphoedema. European Journal of Vascular and Endovascular Surgery 42(2):254–260. 21454105 10.1016/j.ejvs.2011.02.029

[15] Kubo M, Li TS, Kamota T, Ohshima M, Shirasawa B, et al (2010) Extracorporeal shock wave therapy ameliorates secondary lymphedema by promoting lymphangiogenesis. Journal of Vascular Surgery 52(2):429–434. 20670777 10.1016/j.jvs.2010.03.017

[16] Holfeld J, Tepeköylü C, Kozaryn R, Urbschat A, Zacharowski K, et al (2014) Shockwave therapy differentially stimulates endothelial cells: implications on the control of inflammation via Toll-like receptor 3. Inflammation 37(1):65–70. 23948864 10.1007/s10753-013-9712-1

[17] Chen YJ, Kuo YR, Yang KD, Wang CJ, Sheen Chen SM, et al (2004) Activation of extracellular signal-regulated kinase (ERK) and p38 kinase in shock wave-promoted bone formation of segmental defect in rats. Bone 34(3):466–477. 15003794 10.1016/j.bone.2003.11.013

[18] Weihs AM, Fuchs C, Teuschl AH, Hartinger J, Slezak P, et al. (2014) Shockwave treatment enhances cell proliferation and improves wound healing by ATP release coupled extracellular signal-regulated kinase (ERK) activation. Journal of Biological Chemistry, jbc-M114. doi: 10.1074/jbc.M114.580936. PMC4175346 25118288

[19] Marin T, Gongol B, Chen Z, Woo B, Subramaniam S, et al (2013) Mechanosensitive microRNAs—role in endothelial responses to shear stress and redox state. Free Radical Biology and Medicine 64:61–68. 23727269 10.1016/j.freeradbiomed.2013.05.034 PMC3762952

[20] Kriehuber E, Breiteneder-Geleff S, Groeger M, Soleiman A, Schoppmann SF, et al (2001) Isolation and characterization of dermal lymphatic and blood endothelial cells reveal stable and functionally specialized cell lineages. The Journal of Experimental Medicine 194(6):797–808. 11560995 10.1084/jem.194.6.797 PMC2195953

[21] Gröger M, Niederleithner H, Kerjaschki D, Petzelbauer P (2007) A previously unknown dermal blood vessel phenotype in skin inflammation. Journal of Investigative Dermatology 127(12):2893–2900. 17882274 10.1038/sj.jid.5701031

[22] Petzelbauer P, Bender JR, Wilson J, Pober JS (1993) Heterogeneity of dermal microvascular endothelial cell antigen expression and cytokine responsiveness in situ and in cell culture. The Journal of Immunology 151(9):5062–5072. 7691964

[23] Wolbank S, Peterbauer A, Fahrner M, Hennerbichler S, Van Griensven M, et al (2007) Dose-dependent immunomodulatory effect of human stem cells from amniotic membrane: a comparison with human mesenchymal stem cells from adipose tissue. Tissue engineering 13(6):1173–1183. 17518752 10.1089/ten.2006.0313

[24] Holfeld J, Tepeköylü C, Kozaryn R, Mathes W, Grimm M, et al (2014) Shock Wave Application to Cell Cultures. Journal of Visualized Experiments 86:e51076–e51076. 10.3791/51076 PMC4165283 24747842

[25] Holnthoner W, Hohenegger K, Husa AM, Muehleder S, Meinl A, et al (2012) Adipose-derived stem cells induce vascular tube formation of outgrowth endothelial cells in a fibrin matrix. Journal of Tissue Engineering and Regenerative Medicine 10.1002/term.1620 23038666

[26] Rohringer S, Hofbauer P, Schneider KH, Husa AM, Feichtinger G, et al (2014) Mechanisms of vasculogenesis in 3D fibrin matrices mediated by the interaction of adipose-derived stem cells and endothelial cells. Angiogenesis 1–13: 10.1007/s10456-014-9439-0 25086616

[27] Dellago H, Preschitz-Kammerhofer B, Terlecki-Zaniewicz L, Schreiner C, Fortschegger K, et al (2013) High levels of oncomiR-21 contribute to the senescence-induced growth arrest in normal human cells and its knock-down increases the replicative lifespan. Aging Cell 12(3):446–458. 23496142 10.1111/acel.12069 PMC3864473

[28] Smyth GK (2004) Linear models and empirical bayes methods for assessing differential expression in microarray experiments. Statistical Applications in Genetics and Molecular Biology 3(1) 10.2202/1544-6115.1027 16646809

[29] Dweep H, Sticht C, Pandey P, Gretz N (2011) miRWalk–database: prediction of possible miRNA binding sites by “walking” the genes of three genomes. Journal of Biomedical Informatics 44(5):839–847. 21605702 10.1016/j.jbi.2011.05.002

[30] Mittermayr R, Hartinger J, Antonic V, Meinl A, Pfeifer S, et al (2011) Extracorporeal shock wave therapy (ESWT) minimizes ischemic tissue necrosis irrespective of application time and promotes tissue revascularization by stimulating angiogenesis. Annals of Surgery 253(5):1024–1032. 21372687 10.1097/SLA.0b013e3182121d6e

[31] Nishida T, Shimokawa H, Oi K, Tatewaki H, Uwatoku T, et al (2004) Extracorporeal cardiac shock wave therapy markedly ameliorates ischemia-induced myocardial dysfunction in pigs in vivo. Circulation 110(19):3055–3061. 15520304 10.1161/01.CIR.0000148849.51177.97

[32] Cortez MA, Nicoloso MS, Shimizu M, Rossi S, Gopisetty G, et al (2010) miR-29b and miR-125a regulate podoplanin and suppress invasion in glioblastoma. Genes, Chromosomes and Cancer 49(11):981–990. 20665731 10.1002/gcc.20808 PMC5559292

[33] Kuo YR, Wang CT, Wang FS, Yang KD, Chiang YC, et al (2009) Extracorporeal shock wave treatment modulates skin fibroblast recruitment and leukocyte infiltration for enhancing extended skin-flap survival. Wound Repair and Regeneration 17(1):80–87. 19152654 10.1111/j.1524-475X.2008.00444.x

[34] Mäkinen T, Norrmen C, Petrova TV (2007) Molecular mechanisms of lymphatic vascular development. Cellular and Molecular Life Sciences 64(15):1915–1929. 17458498 10.1007/s00018-007-7040-z PMC11136057

[35] Davis TA, Stojadinovic A, Anam K, Amare M, Naik S, et al (2009) Extracorporeal shock wave therapy suppresses the early proinflammatory immune response to a severe cutaneous burn injury. International Wound Journal 6(1):11–21. 19291111 10.1111/j.1742-481X.2008.00540.x PMC7951765

[36] Schacht V, Dadras SS, Johnson LA, Jackson DG, Hong YK, et al (2005) Up-regulation of the lymphatic marker podoplanin, a mucin-type transmembrane glycoprotein, in human squamous cell carcinomas and germ cell tumors. The American Journal of Pathology 166(3):913–921. 15743802 10.1016/S0002-9440(10)62311-5 PMC1602360

[37] Schacht V, Ramirez MI, Hong YK, Hirakawa S, Feng D, et al (2003) T1α/podoplanin deficiency disrupts normal lymphatic vasculature formation and causes lymphedema. The EMBO Journal 22(14):3546–3556. 12853470 10.1093/emboj/cdg342 PMC165612

[38] Gröger M, Loewe R, Holnthoner W, Embacher R, Pillinger M, et al (2004) IL-3 induces expression of lymphatic markers Prox-1 and podoplanin in human endothelial cells. The Journal of Immunology 173(12):7161–7169. 15585837 10.4049/jimmunol.173.12.7161

[39] Cheng M, Guan X, Li H, Cui X, Zhang X, et al (2013) Shear stress regulates late EPC differentiation via mechanosensitive molecule-mediated cytoskeletal rearrangement. PloS One 8(7):e67675. 23844056 10.1371/journal.pone.0067675 PMC3699607

[40] Klein U, Lia M, Crespo M, Siegel R, Shen Q, et al (2010) The DLEU2/miR-15a/16-1 Cluster Controls B Cell Proliferation and Its Deletion Leads to Chronic Lymphocytic Leukemia. Cancer Cell 17(1):28–40. 20060366 10.1016/j.ccr.2009.11.019

[41] Jasielska M, Semkova I, Shi X, Schmidt K, Karagiannis D, et al (2010) Differential role of tumor necrosis factor (TNF)-α receptors in the development of choroidal neovascularization. Investigative Ophthalmology & Visual Science 51(8):3874–3883. 20335614 10.1167/iovs.09-5003

[42] Verma K, Tran D, Bryan BA, Mitchell DC (2013) Meta-analysis of Infantile Hemangioma Endothelial Cell Microarray Expression Data Reveals Significant Aberrations of Gene Networks Involved in Cell Adhesion and Extracellular Matrix Composition. Angiology 1(107):2.

[43] Nagahara A, Nakayama M, Oka D, Tsuchiya M, Kawashima A, et al (2010) SERPINE2 is a possible candidate promotor for lymph node metastasis in testicular cancer. Biochemical and Biophysical Research Communications 391(4):1641–1646. 20035713 10.1016/j.bbrc.2009.12.105

[44] Primo L, di Blasio L, Roca C, Droetto S, Piva R, et al (2007) Essential role of PDK1 in regulating endothelial cell migration. The Journal of Cell Biology 176(7):1035–1047. 17371830 10.1083/jcb.200607053 PMC2064087

[45] Teruyama K, Abe M, Nakano T, Takahashi S, Yamada S, et al (2001) Neurophilin-1 is a downstream target of transcription factor Ets-1 in human umbilical vein endothelial cells. FEBS Letters 504(1):1–4. 11522285 10.1016/s0014-5793(01)02724-7

[46] Rosette C, Roth RB, Oeth P, Braun A, Kammerer S, et al (2005) Role of ICAM1 in invasion of human breast cancer cells. Carcinogenesis 26(5):943–950. 15774488 10.1093/carcin/bgi070

[47] Wick N, Haluza D, Gurnhofer E, Raab I, Kasimir MT, et al (2008) Lymphatic Precollectors Contain a Novel, Specialized Subpopulation of Podoplanin low, CCL27-Expressing Lymphatic Endothelial Cells. The American Journal of Pathology 173(4):1202–1209. 18772332 10.2353/ajpath.2008.080101 PMC2543086

[48] Nisato RE, Harrison JA, Buser R, Orci L, Rinsch C, et al (2004) Generation and Characterization of Telomerase-Transfected Human Lymphatic Endothelial Cells with an Extended Life Span. The American Journal of Pathology 165(1):11–24. 15215158 10.1016/S0002-9440(10)63271-3 PMC1618539

[49] Chang MWF, Grillari J, Mayrhofer C, Fortschegger K, Allmaier G, et al (2005) Comparison of early passage, senescent and hTERT immortalized endothelial cells. Experimental Cell Research 309(1):121–136. 15964568 10.1016/j.yexcr.2005.05.002

[50] Schoppmann SF, Soleiman A, Kalt R, Okubo Y, Benisch C, et al (2004) Telomerase-Immortalized Lymphatic and Blood Vessel Endothelial Cells are Functionally Stable and Retain Their Lineage Specificity. Microcirculation 11(3):261–269. 15280080 10.1080/10739680490425967

[51] Hofbauer P, Riedl S, Witzeneder K, Hildner F, Wolbank S, et al (2014) Human platelet lysate is a feasible candidate to replace fetal calf serum as medium supplement for blood vascular and lymphatic endothelial cells. Cytotherapy 16(9):1238–1244. 24927718 10.1016/j.jcyt.2014.04.009

