# Supplementary material for: Molecular and Cellular Effects of In Vitro Shockwave Treatment on Lymphatic Endothelial Cells
Source: PLoS One. 2014 Dec 11;9(12):e114806. doi: 10.1371/journal.pone.0114806 (PMC4263473; doi:10.1371/journal.pone.0114806)
Supplement: S1 File — Supplementary Information. S1 Figure, Influences of IVSWT on MG63 proliferation. Shockwave treatment with different energy flux densities had no visible effects on MG63 proliferation. S2 Figure, IVSWT-induced changes of EC adhesion and LEC permeability. (A) The ability of LECs to reattach to a fibronectin-coated surface after shockwave treatment was significantly decreased when cells were stimulated on Cytodex-1 microcarrier beads. (B) HUVEC adhesion was not influenced by IVSWT. (C) Quantification of LEC monolayer permeability demonstrates an increase in permeability right after treatment which decays after 4 hours. P-values: *** ≤0.01, ** ≤0.1, * ≤0.5. S3 Figure, IVSWT-mediated influences on in vitro vasculogenesis. (A) Stimulation of HUVEC/ASC co-cultures on day 0 with following incubation for 7 days showed no visible changes in the amount of junctions, tubules, total and mean length of the developed networks. (B) Network stimulation on day 2 after seeding with fixation on day 4 revealed no effect of IVSWT on vasculogenesis. (C) Choosing an EC∶ASC ratio of 1∶0.5 instead of 1∶1 revealed a converse effect to the 1∶1 ratio results (Shown in figure 2). The amount of junctions, tubules and the total length of tubules decreased whereas the mean tubule length increased. Scale bar = 200 µm. P-values: *** ≤0.01, ** ≤0.1, * ≤0.5. S4 Figure, Flow cytometry analyses of LEC and HUVEC marker expression after IVSWT. (A) Cell surface expression of CD31, VE-Cadherin, VEGFR2, VEGFR3 and LYVE-1 on LECs did not change upon IVSWT whereas a significant upregulation of podoplanin was observed. (B) The expression of CD31, VE-Cadherin, CD146, VEGFR2 and Tie-2 on HUVECs did not change significantly after IVSWT. (C) The upregulation of podoplanin on LECs is energy flux density dependent. 0.03 and 0.09 mJ/mm2 suppressed, whereas 0.07 and 0.09 mJ/mm2 increased podoplanin expression. P-values: *** ≤0.01, ** ≤0.1, * ≤0.5. S5 Figure, Gene ontology (GO) Analysis of genes differentially expressed [file pone.0114806.s001.doc]

**Supplementary information**

Figure S1


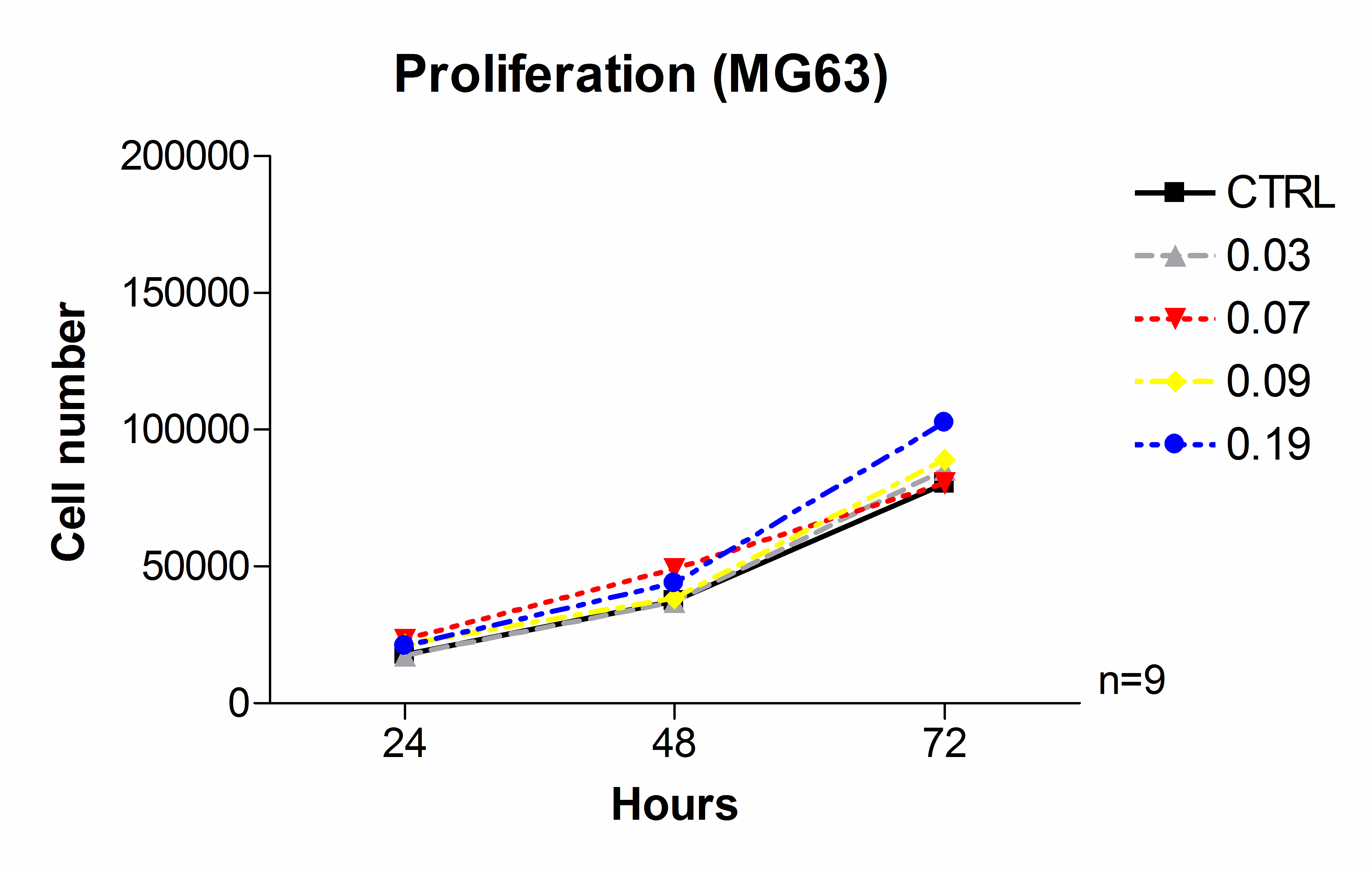


Figure S2


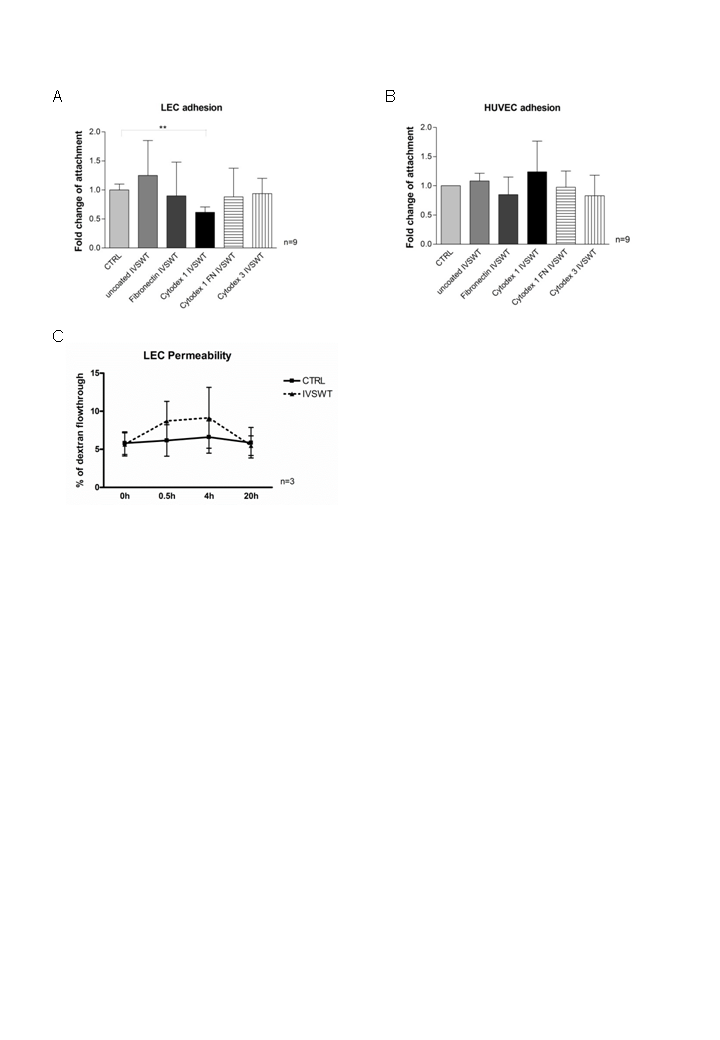


Figure S3


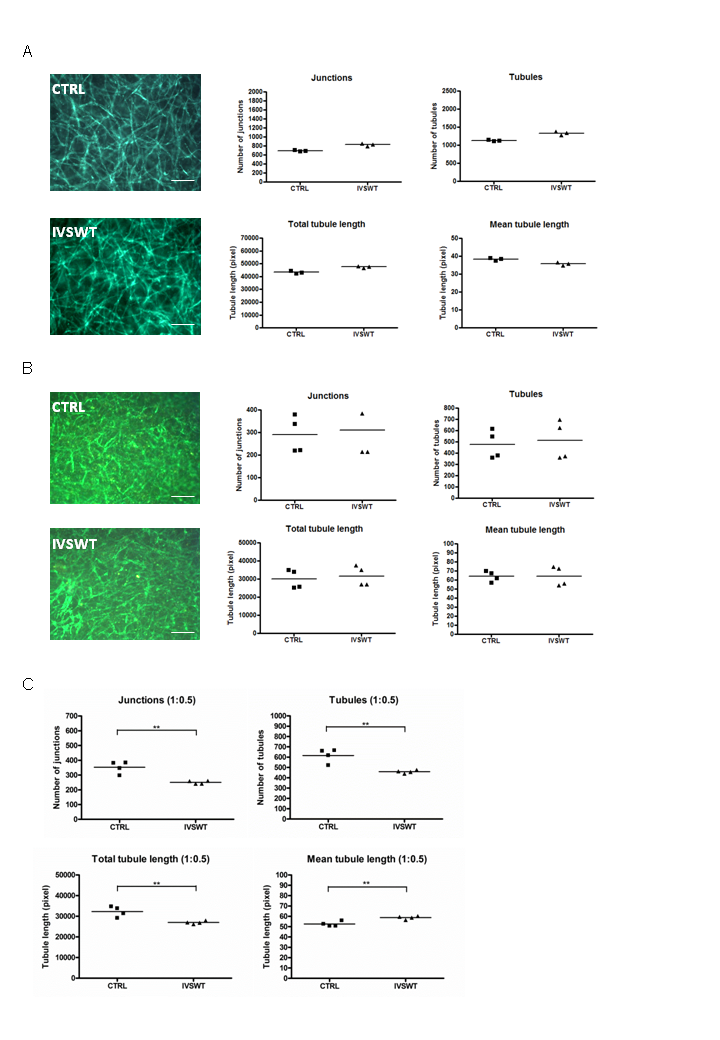


Figure S4


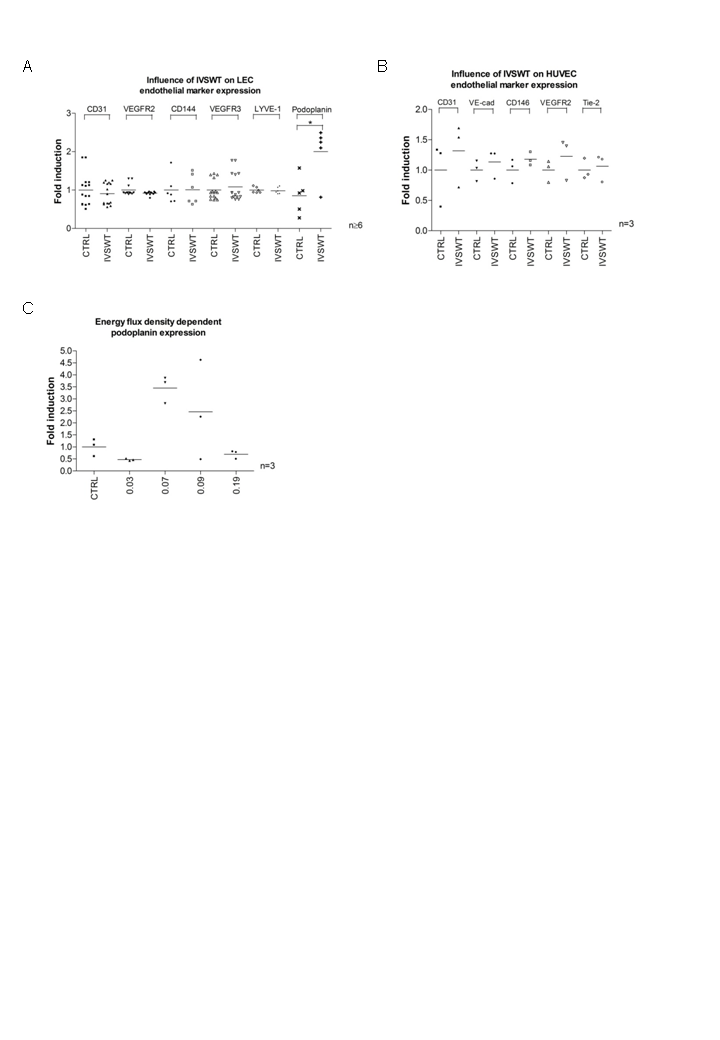


Figure S5


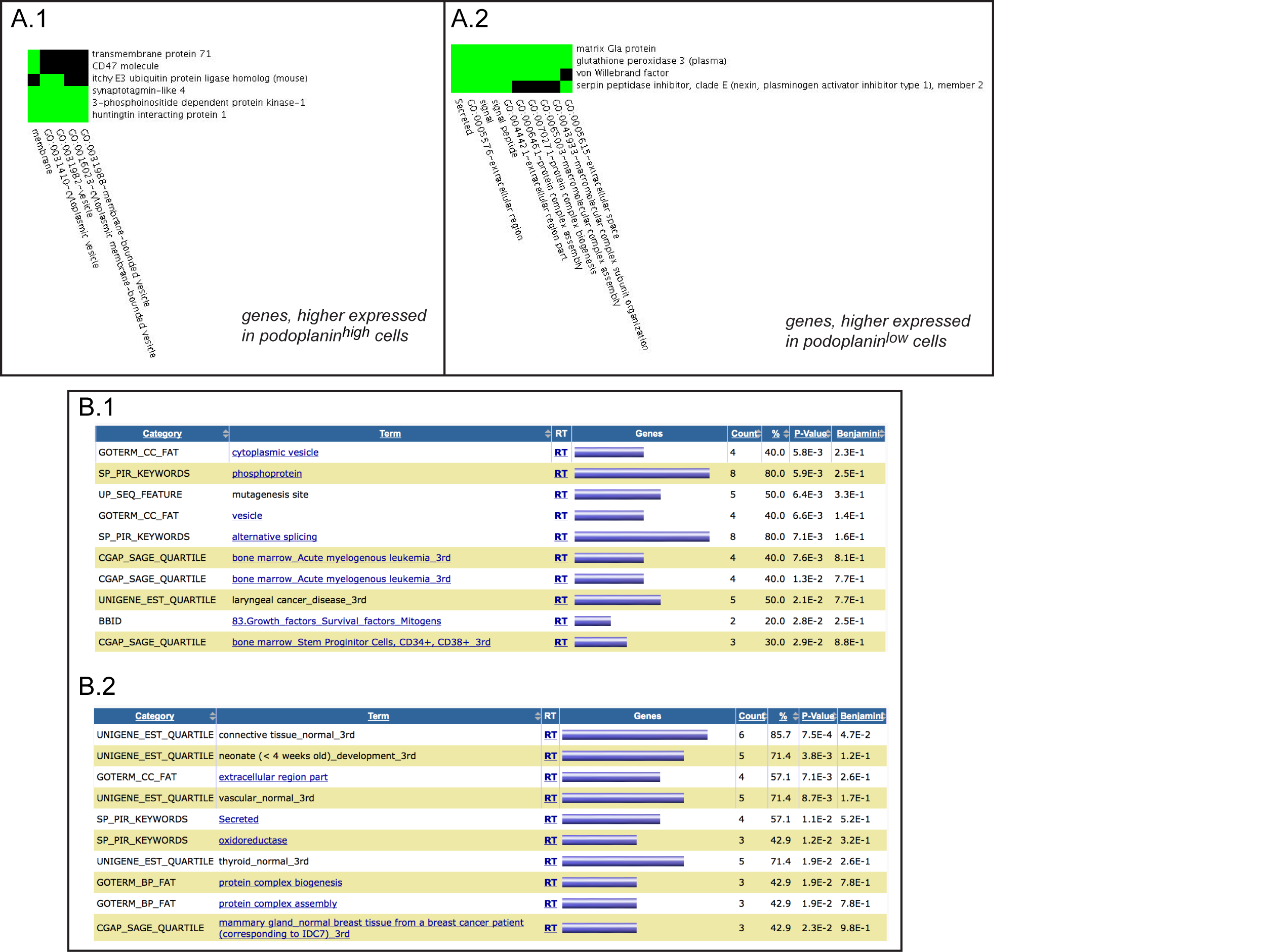


Figure S6


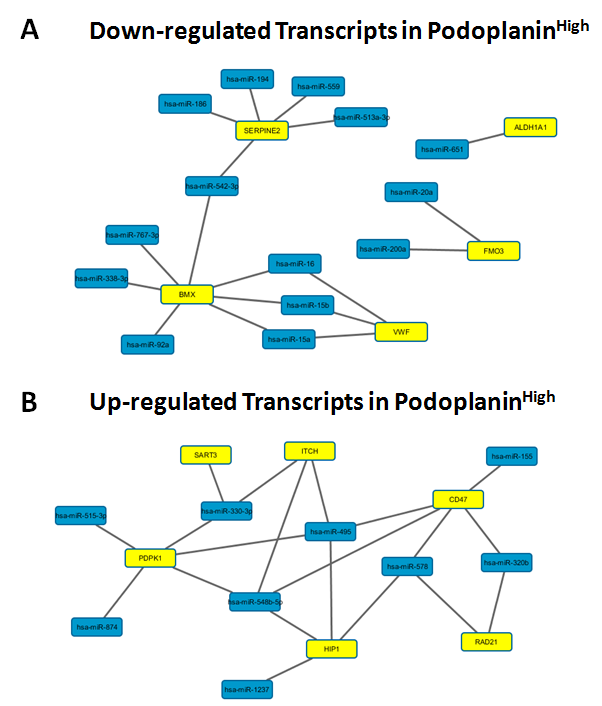


**Supporting Tables**

**TableS1: Predicted microRNA binding sites and correlation analysis in down-regulated genes (Podoplanin^high^ vs Podoplanin^low^)**

| **GENE-ID** | **Predicted miRNA interactions (> 50% of algorithms)** | **Negatively correlated miRNAs (log_2_ FC > 0.5)** | **Positively correlated miRNAs (log_2_ FC < -0.5)** | **miRNAs without changes in transcription** |
| --- | --- | --- | --- | --- |
| **ALDH1A1** | 6 | 1 |  | 5 |
| **BMX** | 15 | 7 |  | 8 |
| **FMO3** | 3 | 2 |  | 1 |
| **SERPINE2** | 9 | 5 | 1 | 3 |
| **VWF** | 10 | 3 |  | 7 |

**Table S2: Predicted microRNA binding sites and correlation analysis in up-regulated genes (Podoplanin^high^ vs Podoplanin^low^**)

| **GENE-ID** | **Predicted miRNA interactions (> 50% of algorithms)** | **Negatively correlated miRNAs (log_2_ FC < -0.5)** | **Positively correlated miRNAs (log_2_ FC > 0.5)** | **miRNAs without changes in transcription** |
| --- | --- | --- | --- | --- |
| **CD47** | 94 | 5 | 24 | 65 |
| **HIP1** | 95 | 4 | 20 | 71 |
| **ITCH** | 65 | 3 | 14 | 48 |
| **PDPK1** | 97 | 5 | 19 | 73 |
| **RAD21** | 37 | 2 | 9 | 26 |
| **RASEF** | 5 |  | 1 | 4 |
| **SART3** | 36 | 1 | 7 | 28 |
| **SYTL4** | 1 |  |  | 1 |
| **TMEM71** | 33 |  | 8 | 25 |
